# Group IIC self-splicing intron-derived novel circular RNA vaccine elicits superior immune response against RSV

**DOI:** 10.3389/fimmu.2025.1574568

**Published:** 2025-04-11

**Authors:** Zeyun Sun, Lirong Lu, Lijie Liu, Ruoxu Liang, Qiqi Zhang, Zhining Liu, Jiahao An, Qian Liu, Qingxin Wu, Shuai Wei, Long Zhang, Wei Peng

**Affiliations:** ^1^ KingMed School of Laboratory Medicine, Guangzhou Medical University, Guangzhou, China; ^2^ Graduate School of Guangzhou Medical University, Guangzhou Medical University, Guangzhou, China; ^3^ Guangzhou National Laboratory, Guangzhou, China; ^4^ School of Pharmaceutical Sciences, Guangzhou Medical University, Guangzhou, China; ^5^ Institute of Pathogenic Biology, School of Basic Medical Sciences, Hengyang Medical School, University of South China, Hengyang, Hunan, China; ^6^ School of Materials Science and Engineering, Sun Yat-Sen University, Guangzhou, China

**Keywords:** circular RNA, group IIC self-splicing intron, RSV, nucleic acid vaccine, PIE, immune response

## Abstract

**Introduction:**

The remarkable commercial success of mRNA vaccines against COVID-19 and tumors, along with their potential as therapeutic drugs, has significantly boosted enthusiasm for circular RNAs (circRNA) as a promising next-generation therapeutic platform. The development of novel circRNA cyclization technologies represents a significant leap forward in RNA engineering and therapeutic applications. Recent advancements in group I and IIB self-splicing intron-based ribozymes have enabled precise cyclization of RNA molecules. However, this approach faces significant limitations, including low cyclization efficiency and the requirement for additional additives, which restrict its broader application. Group IIC self-splicing introns represent the shortest known selfsplicing ribozymes and employ a splicing mechanism that is fundamentally distinct from that of group IIB self-splicing introns. However, the potential of group IIC self-splicing introns to carry exogenous sequences for the development of circular RNA-based platforms remains an open question and warrants further investigation.

**Methods:**

Here, we demonstrate that group IIC self-splicing introns can efficiently circularize and express exogenous proteins of varying lengths, as evidenced by luciferase and GFP reporter systems. Leveraging structural biology-based design, we engineered the RSV pre-F protein and validated the potential of IIC self-splicing introns as a vaccine platform for preventing infectious diseases.

**Results:**

In mouse models, the novel nucleic acid vaccine developed using IIC self-splicing introns elicited superior immunogenicity and in vivo protective efficacy compared to protein-adjuvant vaccines.

**Discussion:**

The development of the novel circular RNA vaccine platform holds significant promise for advancing next-generation therapeutics for disease treatment and prevention.

## 1 Introduction

Respiratory syncytial virus (RSV) is a single-stranded negative-sense RNA virus. It is among the most common causes of acute lower respiratory tract infection in children, older adults, and immunocompromised persons (1–4). The fusion (F) glycoprotein, a trimeric protein anchored on the viral surface, mediates viral fusion and is the major antigen for vaccine development. RSV F exists in two different conformations, the prefusion (pre-F) and the post fusion (post-F) states (5–7). Most of the potent neutralizing antibodies are directed to the pre-F state (8, 9). However, the pre-F state is metastable, readily converting into the more stable post-F state (10, 11). Therefore, arresting RSV F in its pre-F state is a promising strategy for vaccine design (5, 12).

Several approaches have been used to stabilize RSV F in its highly immunogenic pre-F state. The pre-F antigen of GSK’s RSV vaccine called DS-Cav1, which was generated by using a disulfide bond and two cavity-filling substitutions. Based on DS-Cav1 design, an additional disulfide bond was incorporated to generate DS2, which has been used in Moderna’s mRNA vaccine (13). In addition, researchers from both Janssen and Pfizer have used other mutation schemes to achieve stabilization of the RSV pre-F conformation. Another strategy to stabilize its pre-F state was developed by blocking localized changes in protein structure that are coupled with the large-scale conformational rearrangements during the pre-F to post-F conversion (Mutating a flexible region of the RSV F protein can stabilize the prefusion conformation) (14–16).

Currently, the US Food and Drug Administration (FDA) has approved two recombinant protein vaccines for use. mRNA technology has recently gained attention as a promising new therapeutic approach, leading to the rapid development and market licensing of mRNA vaccines (17–21). However, this technology also has notable drawbacks, including the short half-life of mRNA *in vivo*, the necessity for cap reactions during production, and the increased costs associated with additional modified bases and the requirement for ultra-low temperature storage. In contrast, circular RNAs, which are single-stranded and covalently closed, exhibit natural resistance to degradation by exonucleases, resulting in improved stability both *in vitro* and *in vivo* compared to linear RNA molecules (22–24). Utilization of either group I or group IIB self-splicing introns platform, virus-specific circular vaccines demonstrate effective antiviral activity and pave the way for innovative research in future vaccine development (25–28).

Here, we developed a novel IIC self-splicing introns-derived circular RNA platform and evaluate its potential in vaccine development by using a RSV pre-F protein, which was designed based on structural biology. Our method offers a novel platform for circRNA-based vaccine development which has broad application in the new generation of mRNA therapy.

## 2 Result

### 2.1 Prefusion conformation confirmation and immunogenicity evaluation of designed RSV immunogen

**Figure 1:**
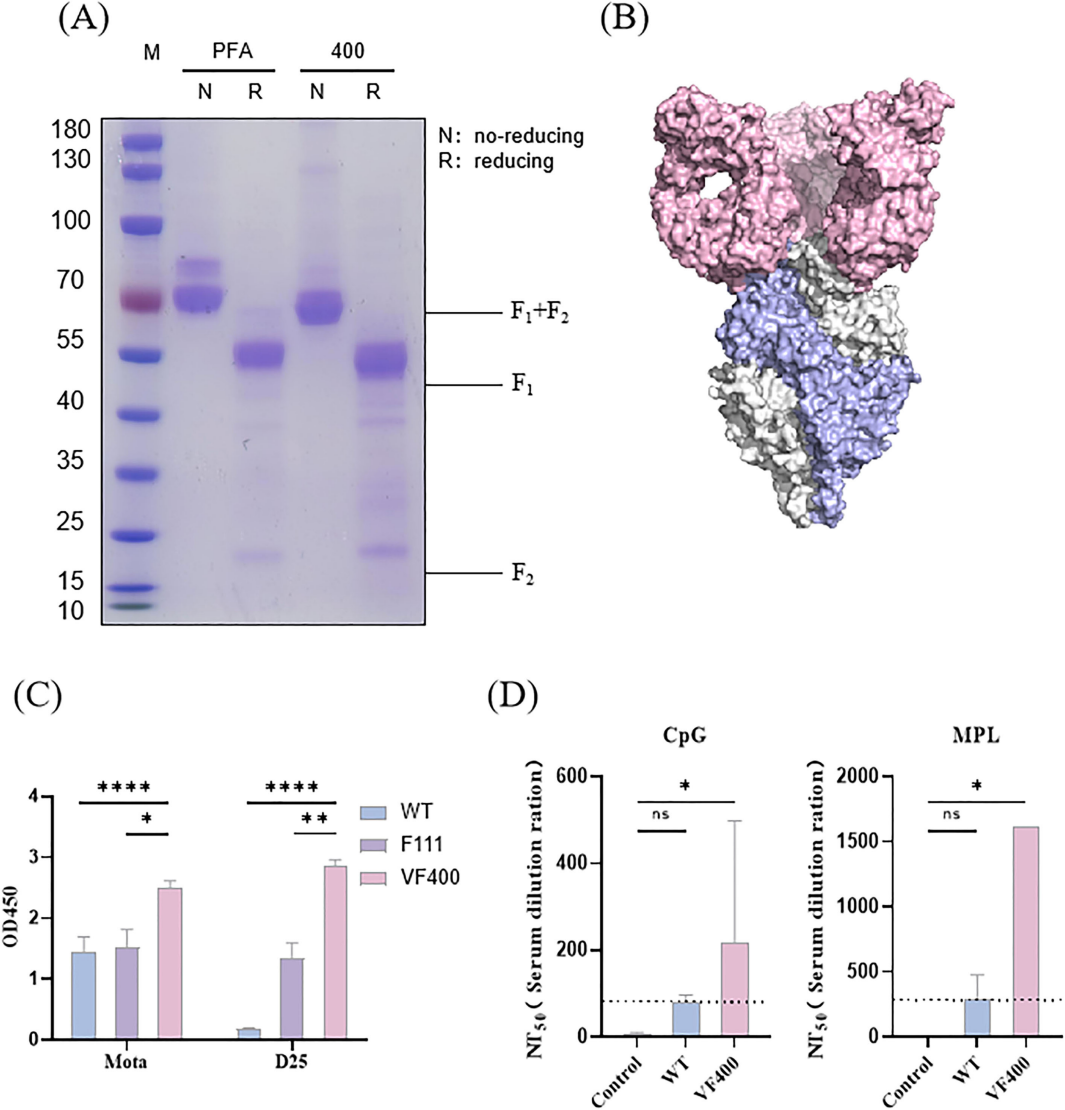
Prefusion conformation confirmation and immunogenicity evaluation of designed RSV immunogen. **(A)** Purified VF400 and PFA were analyzed by SDS-PAGE under reducing (R) and non-reducing (NR) conditions. **(B)** Cryo-EM density map of the prefusion trimer conformation of VF-400 (monomer showed in blue) combined with D25 Fab (pink). **(C)** ELISA detection of Mota and D25 reactivity in the supernatant of HEK 293t cells, assessing the expression of WT (blue), VF400 (purple), and positive control F111 (pink) by detecting the His - tag. **(D)** RSV neutralization titers (NT50) were assessed in mice immunized twice with 0.03 μg VF400 adjuvanted with CpG or MPL, three weeks apart, showing negative control (gray), WT (blue), and VF400 (pink) groups. In **(C, D)**, the height of the bar in the graph indicates the geometric mean calculation for the group ± 95% CI. The significance of differences across groups was assessed by two-sided unpaired T-test with Welch’s correction. *p < 0.05; **p < 0.01; ****p < 0001; ns, not significant.

To develop a candidate vaccine for RSV prefusion F with improved stability in its prefusion conformation and immunogenicity, a strategy combining structural biology design and experimental validation was utilized. The candidate antigen and the control antigen were designated as VF400 and PFA(Pfizer’s antigen), respectively. The designed RSV F protein, which was purified utilizing the AKTA pure™ chromatography system, underwent confirmation through SDS-PAGE analysis. As depicted in **Figure 1A**, both the purified candidate antigen and the control antigen demonstrated high levels of purity. SDS-PAGE analysis was performed under reducing and non-reducing conditions to assess the disulfide bond formations. Under reducing conditions, VF400 and PFA appeared as two bands on the gel, representing the cleaved F1 and F2 fragments, as expected. Under non-reducing conditions, VF400 and PFA appeared primarily as a band near 50 kDa, representing one cleaved F1 fragment (**Figure 1A**).

The structural integrity and prefusion conformation of the RSV F constructs were rigorously assessed utilizing cryoelectronic microscopy (Cryo-EM). The resultant electron density maps elucidated the recombinant pre-F protein VF400, revealing a pre-F trimer exhibiting a pronounced lollipop-like conformation. This structural arrangement is distinguished by the tightly intertwined configuration of three promoters around a threefold axis of symmetry, highlighting the intricate nature of this molecular assembly (**Figure 1B**) (29). By employing two reference monoclonal antibodies (mAbs), its prefusion conformation was further verified by ELISA assay. These two mAbs included prefusion-specific site Ø mAb (D25) and site II-specific mAbs that bind to both pre- and post-fusion F (Mota) (11, 30). The ELISA result revealed the VF400 antigen exhibiting higher prefusion-specific mAb D25 binding than control pre-F antigen F111, suggesting that its conformation is mainly the prefusion conformation (**Figure 1C**) (31).

Immunogenicity is crucial for evaluating vaccine efficacy. This study investigated the immunogenicity of VF400 by immunizing mice twice, three weeks apart, with 0.03 μg of VF400 along with either CpG or MPL adjuvant. The results showed that at this dosage, VF400 significantly increased RSV-neutralizing titers (NT50) compared to the wild-type F protein. Notably, the combination of VF400 with MPL adjuvant produced higher NT50 values than the CpG adjuvant, indicating a stronger immune response (**Figure 1D**).

### 2.2 Design, production and evaluation of IIC self-splicing introns-derived circular RNAs *in vitro*

**Figure 2:**
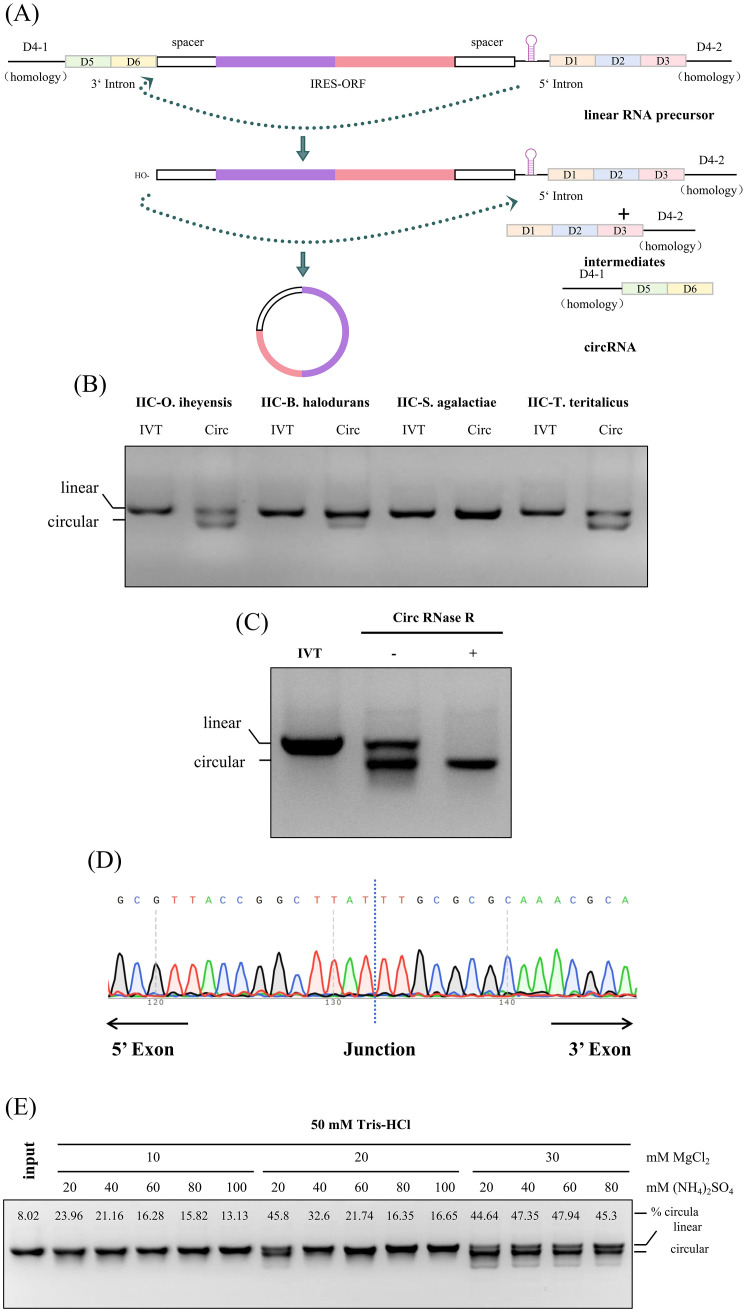
Design, production and evaluation of IIC self-splicing introns-derived circular RNAs *in vitro*. **(A)** Schematic diagram of cyclization. **(B)** 2% agarose gel electrophoresis of RNA from different IIC intron sources, including *O. iheyensis*, *B*. *halodurans*, *S. agalactiae*, and *T. teritalicus*; The loading amount was 1.5 µg, and the gel was run for 35 minutes. **(C)** 2% agarose gel electrophoresis of circular RNA after RNase R digestion. The loading amount was 1.5 µg, and the gel was run for 35 minutes. **(D)** Sanger sequencing of reverse PCR products from purified circular RNA to verify cyclization. **(E)** 2% agarose gel electrophoresis showing the cyclization of RNA at different concentrations of MgCl_2_ and (NH_4_)_2_SO_4_ to determine the optimal cyclization buffer. The circular proportion was calculated by grayscale value analysis using ImageJ.

As the shortest reported type II intron, it is still unknown whether IIC self-splicing introns with exogenous fragments can be cyclized and then express exogenous fragments (32, 33). To efficiently produce IIC self-splicing introns-derived circular RNAs, we employed PIE-based RNA cyclization strategies with modified IIC intron. The autocatalytic self-splicing group IIC intron was split into two fragments at the D4 domain (34), and a spacer followed exons containing IRES and coding region of a gene of interest (GOI,ORF) were inserted between the split intron (35). Upon the *in vitro* transcription, the resulting RNA precursor could produce a circular RNA by a by the two steps self-splicing reaction (**Figure 2A**) (36, 37). It is reported that Group IIC intron can splice via hydrolytic pathway during the first step, while Group IIB intron is via a branching pathway (38, 39).

We initially verified that the group IIC self-splicing intron from a variety of sources, including *-O. iheyensis*, *B. halodurans*, *S. agalactiae*, and *T. teritalicus*, demonstrates self-splicing capabilities in most of strains. This self-splicing process yields about 50% circularized RNA under mild reaction conditions, eliminating the need for additives such as GTP (**Figure 2B**). To determine whether the product is circular RNA, the RNase R digestion was conducted to verify the cyclization efficiency of *O. iheyensis*-derived cyclization reaction products. As shown in **Figure 2C**, circRNAs exhibited resistance to RNase R, whereas their linear RNA counterparts did not (**Figure 2C**). This observation was further supported by Sanger sequencing results obtained using specifically designed reverse PCR primers, which confirmed successful ligation (**Figure 2D**). These findings collectively demonstrate the integrity and functionality of the circular RNA. To increase the cyclization efficiency, the reaction conditions was optimized using different concentrations of MgCl_2_ and NH_4_Cl in the cyclization buffer following the IVT step, and found that using 10× reaction buffer with 300mM Mg^2+^ with 600mM NH_4_^+^ is the optimal reaction condition for the RNA cyclization (**Figure 2E**).

### 2.3 Evaluation of the exogenous protein cyclization efficiency and expression capability of the IIC intron-derived cyclization platform

**Figure 3:**
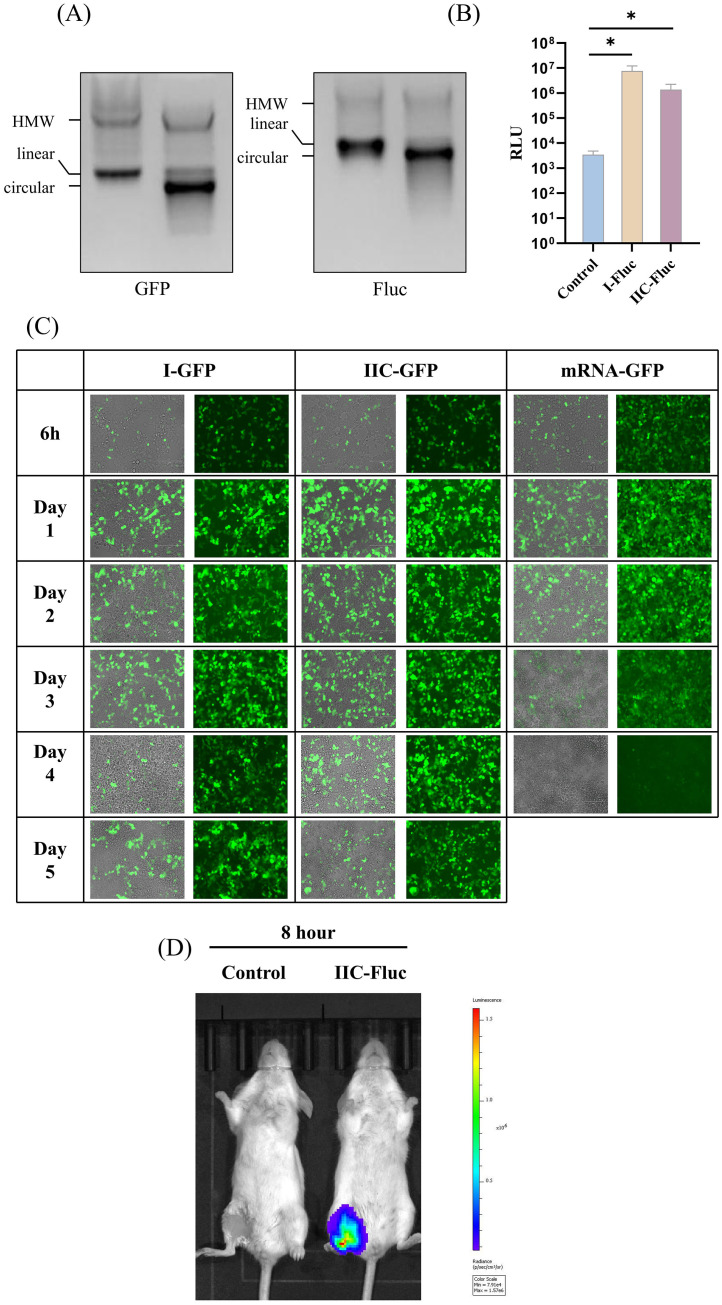
Evaluation of the exogenous protein expression capability of the IIC intron-derived circular platform *in vitro* and *in vivo*. **(A)** 2% agarose gel electrophoresis showing the cyclization of IIC encoding eGFP and Fluc. **(B)** IIC-Fluc transfected HEK 293t cells were collected and lysed 48 hours post - transfection, and the luminescence signal was measured. **(C)** HEK 293t cells were transfected with IIC-GFP, control mRNA, and type I circular RNA; Green fluorescence was observed at 6h, Day 1, Day 2, Day 3, and Day 4 to monitor the expression of GFP. **(D)** Luciferase expression in BALB/c mice after intramuscular injection of LNP-encapsulated IIC-Fluc, as visualized by *in vivo* imaging. In **(C)**, the height of the bar in the graph indicates the geometric mean calculation for the group ± 95% CI. The significance of differences across groups was assessed by two-sided unpaired T-test with Welch’s correction. *p < 0.05.

The efficient circularization of exogenous sequences, particularly long exogenous sequences, is a key technical point and challenge in the development of circular RNA platforms.The exogenous protein cyclization efficiency and expression capabilities of IIC self-splicing intron circular RNA platform were evaluated using either the enhanced Green Fluorescent Protein (eGFP) or Firefly luciferase (Fluc) as the POI. The high cyclization efficiency was confirmed through urea denaturing gel analyses (**Figure 3A**).

Using a fluorescent enzyme detection kit, we verified the expression of Fluc protein in HEK 293T cells 48 hours post-transfection. Compared with the control cell lysate, the cell lysates of the circRNA transfection group showed significantly higher luminescence signals (**Figure 3B**). The IIC-derived circular RNAs were transfected into 293T cells, where eGFP expression was monitored at various time points post-transfection. Notably, green fluorescence was detectable as early as 6 hours post-transfection, and maintaining expression for up to at least 5 days in the circular RNA group, in contrast to the mRNA group (**Figure 3C**). These results demonstrate that the ability of circRNA constructed from the IIC self-splicing ribozyme to express foreign proteins is similar to that of circRNA derived from the type I ribozyme.

To evaluate the capacity for exogenous protein expression *in vivo*, we used lipid nanoparticle (LNP) delivery of IIC-Fluc circRNA. After intramuscular injection, the Fluc circRNAs induced luciferase expression in muscle tissues 8 h post injection (**Figure 3D**).

### 2.4 Construction and characterization of IIC-RSV nucleic acid vaccine *in vitro*

**Figure 4:**
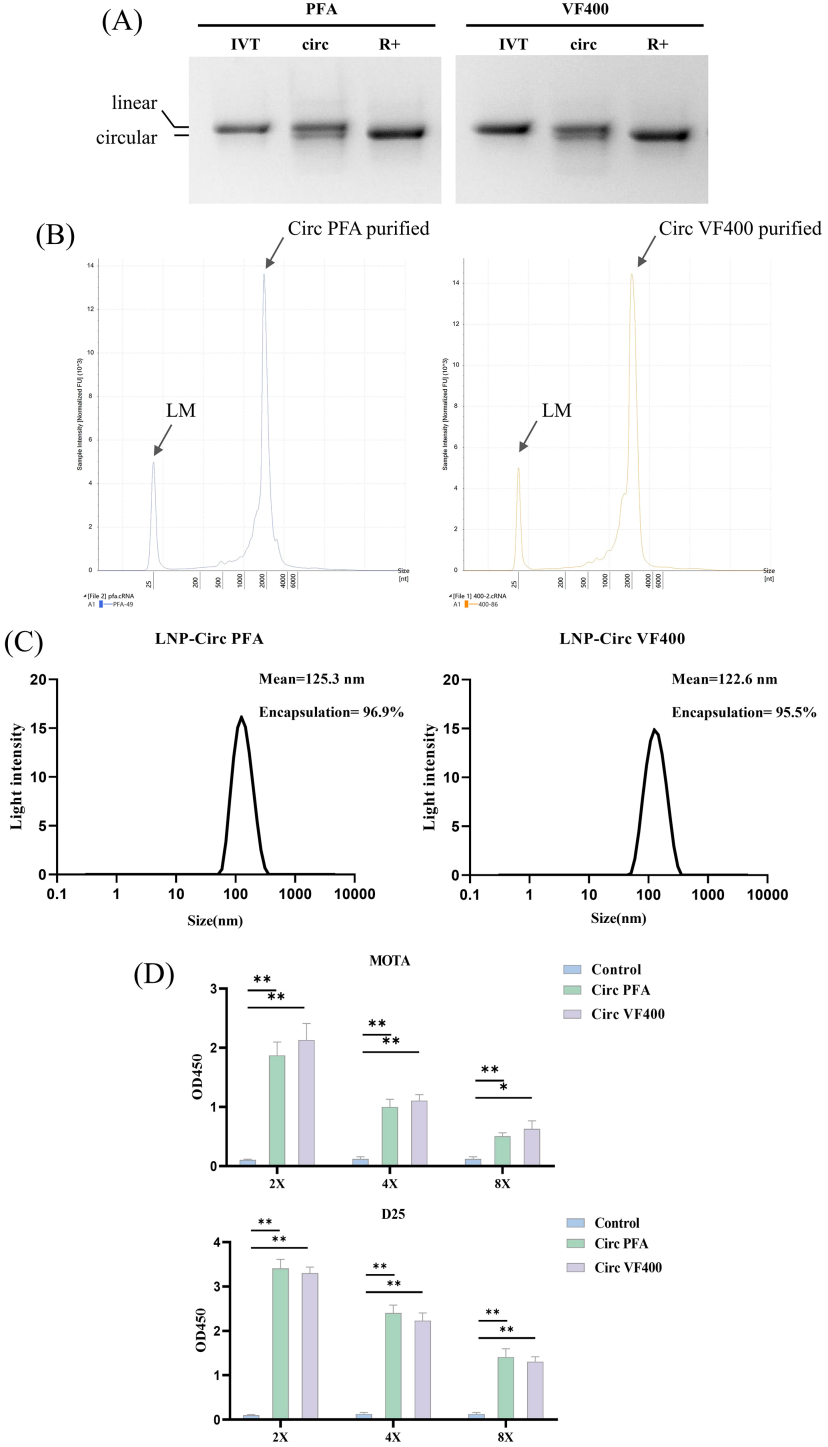
Construction and characterization of IIC-RSV nucleic acid vaccine *in vitro*. **(A)** 2% agarose gel electrophoresis showing the cyclization of IIC capable of expressing VF400 and PFA after RNase R digestion, along with their precursor RNA and circularized products. **(B)** Purity analysis of purified circular RNA VF400 and PFA by capillary electrophoresis (CE). **(C)** Representative intensity-size graph of LNP-circRNA by the dynamic light scattering method. **(D)** ELISA detection of Mota and D25 reactivity in the supernatant of HEK 293t cells, assessing the expression of negative control (blue), Plasmid PFA (red), Plasmid VF400(yellow), CircPFA (green) and Circ VF400 (purple), by detecting the His - tag. In **(D)**, the height of the bar in the graph indicates the geometric mean calculation for the group ± 95% CI. The significance of differences across groups was assessed using one-way ANOVA with Tukey’s test. *p < 0.05; **p < 0.01.

CircRNAs encoding VF400 and Pfizer’s preF (pXCS847, referred to as PFA) were synthesized using the PIE splicing strategy (illustrated in **Figure 2A**). Gel electrophoresis demonstrated that cyclization products after RNase R treatment was shown to have very high purity and can be used for animal immunization (**Figure 4A**). The production of circRNA from RNase R digestion of circulating RNA was subjected to high-performance liquid chromatography (HPLC) for purification. The purity of the purified samples was subsequently assessed using capillary electrophoresis (CE). The CE analysis confirmed that the majority of the resulting products were indeed circRNA, as evidenced by gel electrophoresis results (**Figure 4B**). Following synthesis, these purified circRNAs were encapsulated into LNPs through a microfluidic system, resulting in a ~95% encapsulate efficiency with the effective diameter at ~120 nm (**Figure 4C**). The expression of the preF protein was confirmed using ELISA with the previously mentioned D25 and Mota antibodies (**Figure 1C**). By employing reference antibodies, we established that the F protein expressed by IIC-derived circRNA predominantly adopts the pre-fusion conformation (**Figure 4D**).

### 2.5 Immunogenicity and T cell reponses of circRNA vaccine

**Figure 5:**
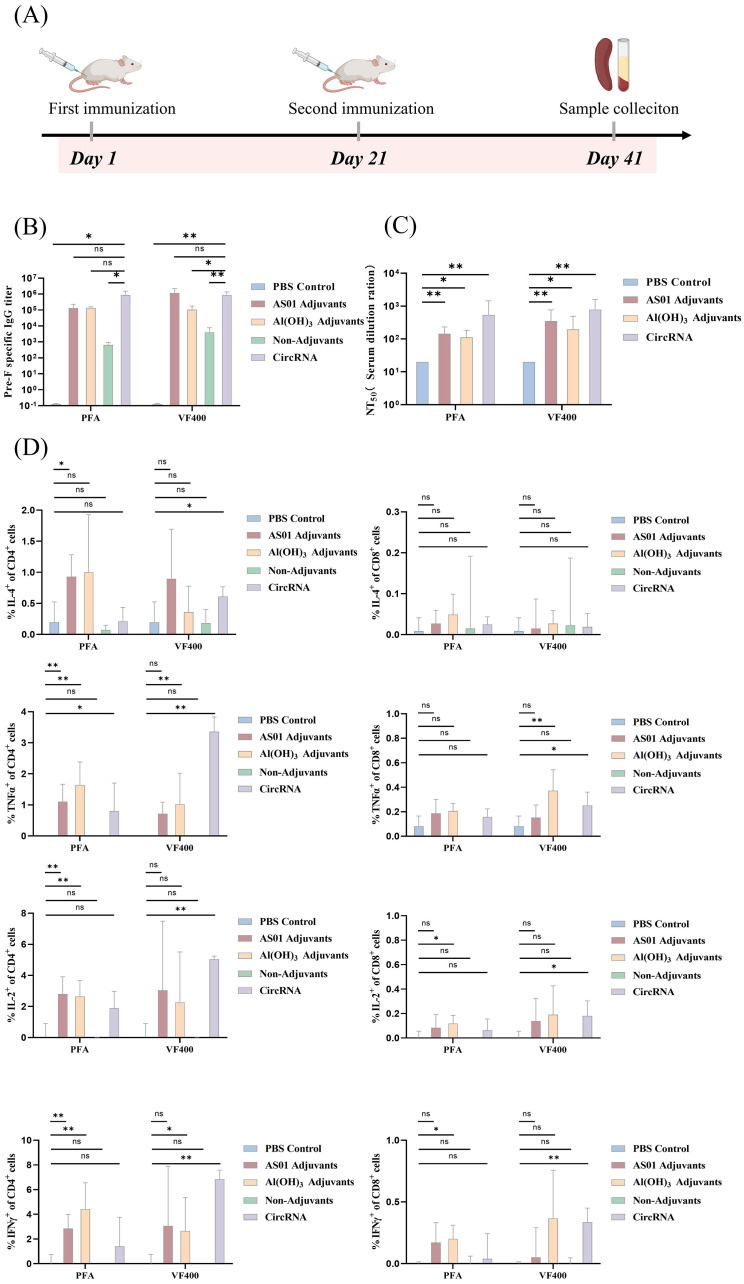
Immunogenicity and T cell reponsed of circRNA vaccine. **(A)** Schematic diagram of the circRNA vaccination and antibody analysis in BALB/c mice. **(B)** Levels of pre-F protein-specific binding antibodies in the circular RNA group (purple) were tested by ELISA, compared with the PBS group (blue), AS01 adjuvant protein group (red), Al adjuvant protein group (yellow), and no adjuvant protein group (green). **(C)** Neutralizing antibody titers against A2 strain in the circular RNA group (purple) were tested by neutralization assay, compared with the PBS group (blue), AS01 adjuvant protein group (red), and Al adjuvant protein group (yellow). **(D)** Spleens were harvested 3 weeks following the second immunization.Splenocytes were incubated with pooled RSV F peptides and were then stained with antibodies to the cell surface markers CD3, CD4, and CD8, as well as with a panel of anti-cytokine antibodies. The percent of CD4+ T-cells and CD8+ T-cells responding to RSV F peptides with production of IFN-γ, IL-2, IL-4 or TNF-α is shown. In **(B–D)**, the height of the bar in the graph indicates the geometric mean calculation for the group ± 95% CI. The significance of differences across groups was assessed by two-sided unpaired T-test with Welch’s correction. *p < 0.05; **p < 0.01; ns, not significant.

Mice were immunized twice with circRNA (20 µg/mouse) or purified protein (0.25µg/mouse), with a three-week interval between immunizations (**Figure 5A**). Additionally, some groups received the protein mixed with the adjuvants AS01 or Al (OH)_3_ (40). Compared to the PBS control group, both circRNA and protein immunizations significantly stimulated the production of high levels of preF protein-specific binding antibodies (**Figure 5B**). Nucleic acid vaccines induced significantly higher levels of binding antibodies when compared to recombinant protein vaccines, with results aligning closely with those seen with adjuvanted protein vaccines (**Figure 5B**). Notably, immunization using circRNA resulted in elevated 50% RSV-neutralizing titers (NT_50_), surpassing the titers achieved with protein combined with the adjuvants AS01 or Al (OH)_3_ (**Figure 5C**). These findings highlight the potential of nucleic acid vaccines in eliciting robust immune responses.

We aimed to measure the T-cell responses elicited by immunization with our circRNA and recombinant protein vaccine candidates. In this study, we analyzed CD4^+^ and CD8^+^ T-cells for the production of key cytokines, including IL-2, IL-4, the antiviral cytokine IFN-γ, and tumor necrosis factor (TNF)-α, to delineate antigen-specific T cell subsets. Our analysis revealed that immunization with circ400 and protein combined with the adjuvant resulted in a significantly higher percentage of CD4^+^ IFN-γ^+^ and CD8^+^ IFN-γ^+^ cells compared to the PBS or protein-only groups (**Figure 5D**).

While the induction of IFN-γ is often used as a parameter for measuring vaccine-specific T-cell immunity (41). Conversely, the production of IL-2 has been shown to be critical for the maintenance of memory T-cells (42). Strikingly, our results indicate that upon incorporation of pre-F antigen into the IIC-circRNA or the protein combined with the adjuvant, there was an elevated CD4^+^ and CD8^+^ IL-2^+^ response compared to both PBS and protein-only groups.

Additionally, we observed CD4^+^ and CD8^+^ TNF-α^+^ responses following IIC-circRNA or protein combined with the adjuvant immunization. Notably, lower levels of Th2-type IL-4^+^ T cells were detected in the spleens of mice from the circRNA, protein-only, and PBS groups (**Figure 5D**). Finally, no significant differences were observed in CD4^+^ and CD8^+^ TNF-α^+^ cells between any of the adjuvant groups and the PBS control.

These findings contribute to our understanding of the T-cell responses elicited by our vaccine candidates and highlight the importance of both cytokine profiles in evaluating vaccine efficacy.

### 2.6 CircRNA vaccine can protect the immunized mice from RSV virus infection

**Figure 6:**
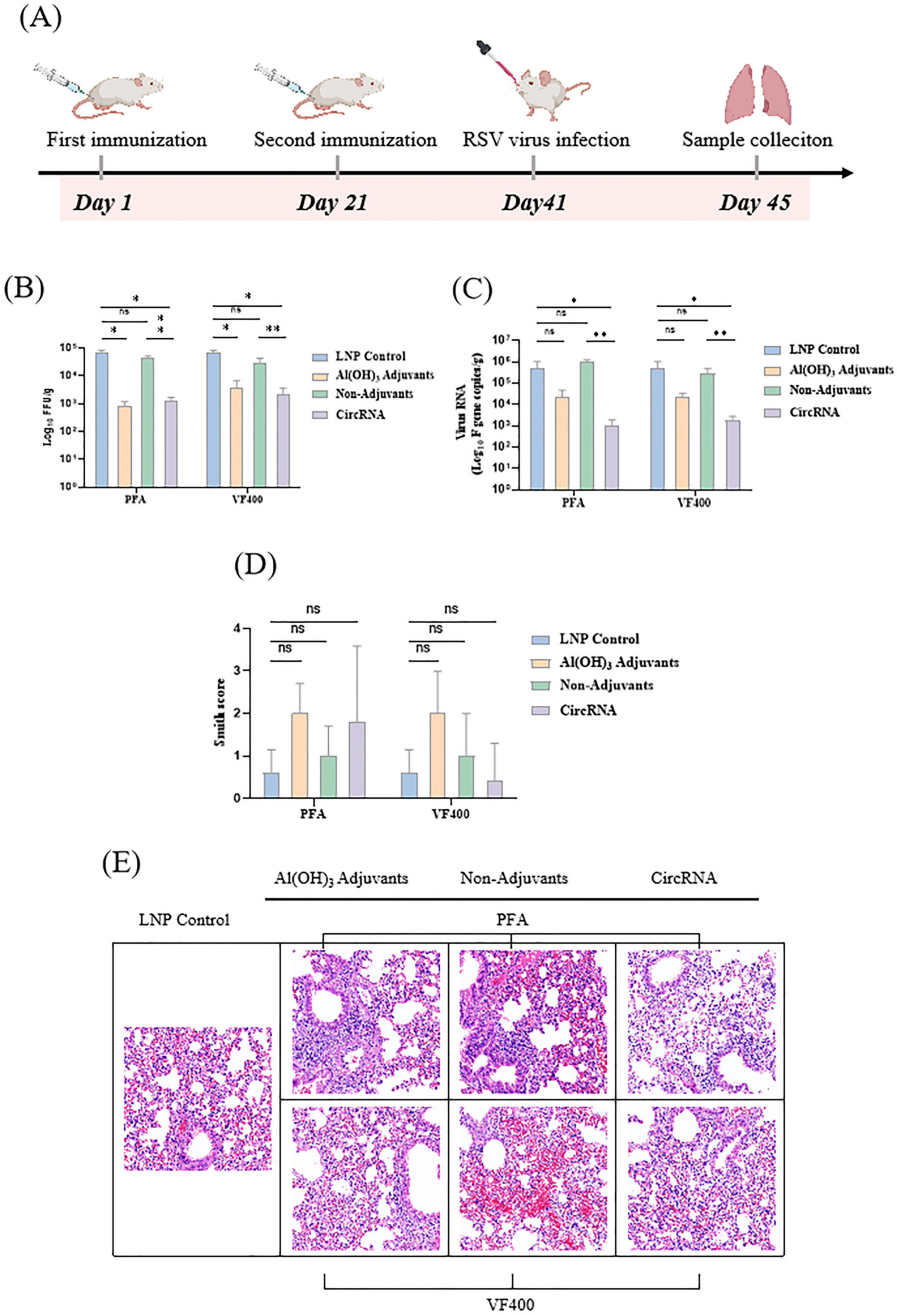
circRNA vaccine can protect the immunized mice from RSV virus infection. **(A)** BALB/c mice were vaccinated twice, with an interval of 3 weeks in between, with proteins or circRNA or only PBS and then challenged with RSV A2 virus at 3 weeks after vaccination. On day 4 after the challenge, the animals were euthanized and lung tissues were harvested. **(B)** Measurement of viral loads in BALB/c mouse lung tissues by focus forming assay (FFA). **(C)** Measurement of the viral loads in BALB/c mouse lung tissues by quantitative polymerase chain reaction (qPCR). **(D)** H&E staining of pathological sections using the lung tissues from immunized BALB/c mice at 4 days after challenge, followed by Smith scoring. The scoring was based on the severity of lung edema, alveolar and interstitial inflammation, alveolar and interstitial hemorrhage, atelectasis, and hyaline membrane formation, with scores ranging from 0 to 4: 0, no damage; 1, lesion extent and area <25%; 2, lesion extent and area: 25% - 50%; 3, lesion extent and area: 50% - 75%; 4, lesion extent and area >75%. **(E)** H&E staining of pathological sections using the lung tissues from immunized BALB/c mice at 4 days after challenge. In **(B–D)**, the height of the bar in the graph indicates the geometric mean calculation for the group ± 95% CI. The significance of differences across groups was assessed using one-way ANOVA with Tukey’s test in **(B)** and **(C)**. The significance of differences across groups was assessed by two-sided unpaired T-test with Welch’s correction in **(D)**. *p < 0.05; **p < 0.01; ns, not significant.

Based on the results obtained, our study indicates that the circRNA vaccine effectively stimulates both Th1-biased cellular and humoral immune responses *in vivo*. To assess the protective efficacy of the IIC intron-derived nucleic acid vaccine, BALB/c mice were immunized bi-weekly and subsequently challenged intranasally with 2 × 10^5^ plaque-forming units (pfu) of the RSV A2 strain. Four days post-infection, lung tissue was collected for viral load and pathological analysis (**Figure 6A**).

We utilized both focus forming assay (FFA) and quantitative polymerase chain reaction (qPCR) to accurately quantify the viral load in the supernatants of lung homogenates. Compared to the LNP group, mice immunized with the circRNA vaccine, protein along with Al (OH)_3_ adjuvant, demonstrated a significant reduction in pulmonary live virus titer (**Figure 6B**) and viral RNA copy number (**Figure 6C**). These findings highlight the promising potential of circRNA vaccines as a protective strategy against RSV infection.

Lung pathology was assessed by scoring for alveolitis, in conjunction with peribronchiolitis, perivasculitis, and interstitial pneumonia. Following immunization with circRNA and subsequent challenge with RSV, no significant differences in lung pathology were observed among different groups (**Figure 6D**). The challenged mice demonstrated typical moderate pathological changes in the lungs, characterized by the presence of thickening of the alveolar walls, inflammation, and hemorrhage. (**Figure 6E**).

## 3 Discussion

The advent of the COVID-19 pandemic has accelerated the development of mRNA vaccines, establishing them as a primary platform for vaccine innovation (43, 44). Presently, numerous mRNA vaccines have received clinical approval (45). In comparison to mRNA technology, circular RNA (circRNA) emerges as an innovative vaccine platform (46), exhibiting a closed-loop structure that confers inherent resistance to nuclease degradation, thereby enhancing stability and extending half-life both *in vivo* and *in vitro*, which also confirmed by our new IIC ribozyme cyclization system (47). Compared with mRNA platform, circGFP based on group I and IIC ribozymes has a longer expression time *in vitro* (**Figure 3C**). This characteristic is particularly advantageous for vaccine storage and transport. Furthermore, the circRNA structure, devoid of free ends, may mitigate the risk of recognition as foreign RNA within cellular environments, thus potentially reducing the incidence of immune suppression reactions. The synthesis of circRNA does not necessitate a 5’ cap or poly(A) tail, nor does it involve the complexities of base modification, rendering its manufacturing process more straightforward and cost-effective (48).

To date, the *in vitro* preparation of circRNA predominantly relies on self-splicing platforms based on the PIE strategy, utilizing type I or type IIB introns. Published studies employing the circRNA platform include vaccines for COVID-19, Zika virus, monkeypox virus, and influenza virus (25–28). Group I and II introns represent a major class of ribozymes capable of undergoing splicing via self-catalytic reactions. Similarly, Group IIC introns facilitate self-splicing through a two-step self-catalytic reaction (49). Currently, it is only established that group IIC introns can execute self-splicing functions. Here, we assessed the viability of *in vitro* cyclization of group IIC introns based on the PIE cyclization approach, and screened various group IIC introns from diverse sources to develop an efficient *in vitro* cyclization platform capable of achieving cyclization of exogenous genes. Consequently, circRNA of group IIC introns presents a promising candidate for RSV vaccine development.

Respiratory syncytial virus (RSV) is a predominant pathogen responsible for lower respiratory tract infections and significant hospitalization rates in infants (50). The development of a safe and efficacious RSV vaccine is of paramount importance to mitigate hospitalization and mortality rates among this vulnerable demographic (51). Despite the ongoing efforts in vaccine and therapeutic development, only three RSV vaccines have attained approval thus far (52). Notably, the prefusion conformation of the RSV F protein is instrumental in eliciting a robust neutralizing antibody response (53–55). Therefore, current RSV vaccine development predominantly focuses on antigenic design, particularly the optimization of the F protein for enhanced stability in its prefusion form (56, 57).

Here, the positive control antigen is of Pfizer’s design, which involved the introduction of disulfide bonds, the filling of hydrophobic cavities, and the incorporation of charge mutations (17). In contrast, our design employed a distinct strategy relying solely on multiple point mutations to fill hydrophobic cavities, aiming to introduce nonpolar amino acids that stabilize the prefusion F protein by inhibiting conformational rearrangements. Utilizing cryo-electron microscopy and antibody binding assays, we demonstrated that the designed antigen preserved its prefusion conformation while maintaining the integrity of the principal antigenic epitopes (**Figure 1A, B**).

Our developed group IIC intron-derived circular RNA platform for expressing RSV pre-F candidate antigen VF400 represents a novel vaccine strategy. As circular RNA vaccines have not yet reached clinical approval whereas recombinant protein vaccines constitute an established approach, this study focused on comparative evaluation with the protein platform. The selected immunization dose of 20 μg LNP-formulated circRNA aligns with published murine protocols recommending 10-50 μg ranges, with 20 μg chosen as a conservative intermediate value balancing efficacy and safety (28). The 0.25 μg protein dose was determined based on positive control data from Pfizer studies demonstrating optimal RSV-neutralizing titers (50% neutralization threshold) in BALB/c mice (58).

Compared to Type I introns, Type IIC introns demonstrated superior expression duration in 293T cells (**Figure 3C**), though current observations may be constrained by adherent cell culture limitations. We hypothesize that organoid cell cultures could better reveal the full persistence potential of Type IIC introns. Type I introns from T4 bacteriophage or by Anabaena pre-tRNA not only require exogenous GTP supplementation during circularization but also introduce ~74 nt td or ~186 nt Anabaena extraneous fragments, which may amplify immunogenicity and activate host recognition pathways and induce immune response (59), thereby potentially suppressing *in vivo* translation. In contrast, Type IIC introns circumvent both limitations. Type IIC intron-based circularization eliminates GTP dependency while retaining merely ~6 nt of highly humanized exonic sequence, thereby minimizing exogenous sequence recognition. This streamlined circularization process offers advantages in manufacturing scalability and cost control through reduced reaction complexity and exogenous additives. Though significant progress has been made in optimizing Type I intron circularization (e.g., single-step or scarless methods), we are exploring novel circularization strategies for Type IIC introns, including co-transcriptional circularization in *in vitro* transcription step. The Type IIC platform presents unique advantages for future vaccine development, providing researchers with an alternative circular RNA scaffold for further exploration and optimization.

In summary, we have developed a new group II introns system for efficient production of circRNAs, which is suitable for vaccine development. The resulting circRNAs can be engineered to direct robust protein translation, providing a new platform of mRNA vaccine with improved stability and antiviral effect. The continuous improvement of this platform should help to take circRNA technology into various clinic applications in the near future.

## 4 Method

### 4.1 Preparation of VF-400 and D25 fab complex and Cryo-EM GridsCryo-EM data collection

To assemble the VF-400 and D25 Fab complex, highly purified proteins were first thawed and then mixed at an equimolar ratio of antigen to antibody (1:1). The mixture was incubated at 4°C for 30 minutes to allow for complex formation. For grid preparation, 2.5 μL of the VF-400 and D25 Fab complex at a concentration of 1 mg/mL was applied to untreated Quantifoil R1.2/1.3 300-mesh copper grids. The samples were then vitrified using a Vitrobot Mark IV robot under controlled conditions: 8°C temperature, 100% relative humidity, a blot force of -3, and a blotting time of 3 seconds. The vitrification process was carried out in liquid ethane to ensure optimal sample preservation for cryo-electron microscopy.

### 4.2 Cryo-EM data collection

Electron cryomicroscopy (Cryo-EM) analysis was performed using a Titan Krios electron microscope operating at an accelerating voltage of 300 keV. The microscope was equipped with a Gatan Falcon 4i direct electron detector, which was operated in super-resolution mode. Imaging was conducted at a magnification of 165,000×, corresponding to a magnified pixel size of 0.73 Å at the specimen level. During data acquisition, the defocus range was set between -0.8 and -2.0 μm, as estimated by the contrast transfer function (CTF), with a total electron dose of 50 e^−^/Å². Image acquisition was controlled using EPU software (Thermo Fisher Scientific).

### 4.3 Data processing and 3D reconstruction

The collected micrographs were initially imported into cryoSPARC for motion correction, CTF estimation, 2D classification, ab initio 3D reconstruction, heterogeneous 3D refinement and non-uniform homogeneous refinement (60). Among the six resulting 3D reconstruction classes, the best-resolved class, containing 14,270 particles, was selected for high-resolution refinement under C3 symmetry, yielding a final resolution of 3.26 Å.

### 4.4 Model building and refinement

A rigid-body fit of the DSCAV-derived crystal structure PDB model (PDB: 4JHW) was performed against the reconstructed density of the F protein-D25 Fab complex. Manual model adjustments were iteratively performed using Coot (61), followed by real-space refinement in Phenix (62) to ensure optimal agreement between the model and density map.

### 4.5 Plasmid construction

The group II self-splicing intron fragments from Marine Bacillus and the IRES sequences were chemically synthesized by Genscript. Different protein - coding fragments (ORFs) and corresponding components were amplified via PCR and then merged with the IRES fragments through the method of homologous recombination (Vazyme). These merged fragments were subsequently cloned into the backbone that had been digested with XbaI, which contained the T7 RNA polymerase promoter and terminator. After that, the RSV pre - F antigen, EGFP, or Fluc was amplified by PCR and cloned into the circRNA - PVAX1 backbone, resulting in the construction of the corresponding circRNA plasmids for use in subsequent *in vitro* transcription (IVT) reactions.

### 4.6 RNA synthesis and circularization

The plasmid DNAs were linearized by digestion with XbaI and purified using a Gel Extraction Kit from Omega. The linearized DNAs served as templates for *in vitro* transcription with the T7 High Yield RNA Synthesis Kit (Yeason) in the presence of unmodified NTPs. Following IVT, the RNA products were treated with DNase I (Thermo Scientific) for 20 minutes to digest the DNA templates. After DNase I treatment, the RNA products were purified using LiCl (Invitrogen) to remove excess NTPs, other salts from the IVT buffer. The purified RNAs were then circularized in a new circularization buffer. The RNAs were initially heated to 70°C for 5 minutes and then quickly cooled to 4°C. After cooling, a buffer containing the specified magnesium and sodium ions was added to achieve the following final concentrations: 50 mM Tris-HCl (pH 7.5), 60 mM (NH_4_)_2_SO_4_, and 30 mM MgCl_2_. The mixture was then heated at 55°C for 15 minutes to facilitate circularization.

### 4.7 CircRNA identification

For RNase R digestion, the RNAs obtained from IVT were purified using LiCl. The purified RNAs were heated at 70°C for 3 minutes, then immediately cooled on ice, and subsequently digested with RNase R exoribonuclease (Geneseed) following the manufacturer’s instructions at 37°C for 15 minutes. The enriched circRNAs were further purified using a column (ZYMO RESEARCH). The RNAs were analyzed by agarose-gel electrophoresis after adding RNA loading dye (NEB). To analyze the circRNA through sequencing, the purified RNA was reverse transcribed into cDNA using a Prime Script RT Reagent Kit with random primers (TaKaRa), followed by PCR amplification with primers designed to amplify transcripts across the splice junction. The PCR products were then subjected to Sanger sequencing to validate the back-spliced junction of the circular RNA.

### 4.8 HPLC purification and electrophoresis of RNA

To obtain high - quality circRNA, the purified RNase R - treated RNA was analyzed using high - performance liquid chromatography (HPLC). For large - scale preparation with EasySepR - 3030 (UNIMICRO), 1 mg of RNA was loaded per run onto a 250 × 20 mm SEC column (Thermo Scientific, BIOBASIC SEC1000). The mobile phase contained 150 mM PB buffer at pH 7.0, and the temperature was maintained at 25°C with a flow rate of 3 ml/min. Fractions were collected as needed and analyzed using agarose gel electrophoresis. The circRNA - enriched fractions were collected and then purified using a column. Circular RNAs purified from large - scale production were further analyzed using capillary electrophoresis with the Agilent 4200 Bioanalyzer in RNA mode. Samples were diluted to an appropriate concentration and analyzed according to the manufacturer’s instructions.

### 4.9 CircRNA transfection *in vitro*

HEK293T or BHK cells were seeded into 12 - well plates one day prior to transfection. Two micrograms of circRNA were transfected using the mRNA Transfection Reagent (Yeason) following the manufacturer’s instructions. After transfection, the cells were incubated at 37°C for 24 - 48 hours, after which cell lysis and the supernatant were collected for subsequent analysis.

### 4.10 LNP encapsulation of circRNA

LNP delivery was formulated using the Moderna recipe. An aqueous solution of circRNA at pH 4.0 was rapidly mixed with a lipid mixture dissolved in ethanol, which contained different ionizable cationic lipids, distearoylphosphatidylcholine (DSPC) (Nippon Fine Chemical Co., Ltd.), DMG-PEG2000 (AVT), and cholesterol, with a molar ratio of SM - 102 (Target Mol): DSPC: Cholesterol: PEG2000 = 50:10:38.5:1.5. For encapsulation, the Blue Magpie microfluidic encapsulator with the Feather Microfluidic Chip (APE×BIO RM1002 - 1) was used. The size of LNP - circRNA particles was measured using dynamic light scattering on a Malvern Zetasizer Nano - ZS 300 (Malvern). Samples were irradiated with a red laser, and the scattered light was detected. The results were analyzed to obtain an autocorrelation function using the software Zetasizer V7.13.

### 4.11 Mouse vaccination and serum collection

For mouse vaccination, groups of 6 - to 7 - week - old female BALB/c mice were intramuscularly immunized with 20 µg of circRNA diluted in 50 µl of 1X PBS using a 1 - ml sterile syringe. For the protein formulation groups, each mouse was administered a dose of 0.25 μg per animal. The AS01 adjuvant was used at a concentration of 50 μg/mL, while the Al(OH)_3_ adjuvant was administered at a dose of 50 μg per mouse. Two weeks later, a second dose was administered to the same hind leg to boost the immune response. Mice in the control groups received either PBS or empty LNPs. Blood samples for detecting the pre - F - specific IgG titer and neutralizing antibodies, as well as mouse spleens for immunostaining and flow cytometry, were collected three weeks after the booster shots.All animal experiments were approved by the Ethics Committee of Guangdong Laboratory Animals Monitoring Institute (GZLAB - AUCR - 2024 - 01 - A6).

### 4.12 Mota and D25 reactivity in the supernatant by Elisa

96 - well flat - bottom plates (CORNING) were incubated with D25 or MOTA antibody in coating buffer (1 µg/mL, 100 µL/well) (Solarbio) at 4°C overnight. The plates were washed three times with PBS - T (Biosharp) and then incubated with Block buffer (2% BSA (ABCONE) in PBS - T) at 37°C for 2 hours. After five washes with PBS - T, thawed cell supernatants were added to the wells at 1/2, 1/4, or 1/8 dilutions (100 µL/well). Following incubation at 37°C for 2 hours, the plates were washed five times with PBS - T and incubated with HRP 6 * His, His - tag antibody (Proteintech) according to the manufacturer’s recommendations. After 1 hour of incubation, the plates were washed five times with PBS - T and incubated with TMB Single - Component Substrate solution (100 µL, Solarbio). Color development was stopped after 15 minutes with Elisa Stop Solution (Solarbio), and the plates were read in a Thermo ScientificTM VarioskanTM LUX at a 450 nm wavelength.

### 4.13 Levels of preF protein-specific binding antibodies by Elisa

96 - well flat - bottom plates (CORNING) were incubated with protein PFA antibody in coating buffer (2 µg/mL, 100 µL/well) (Solarbio) at 4°C overnight. The plates were washed three times with PBS - T (Biosharp) and incubated with Block buffer (2% BSA (ABCONE) in PBS - T) at 37°C for 2 hours. After five washes with PBS - T, the serum was diluted starting from a 1:100 ratio, with 8 concentration gradients in a 10 - fold dilution series. Following incubation at 37°C for 2 hours, the plates were washed five times with PBS - T and incubated with Goat Anti - Mouse IgG (H+L) HRP (GLGBIO) according to the manufacturer’s recommendations. After 1 hour of incubation, the plates were washed five times with PBS - T and incubated with TMB Single - Component Substrate solution (100 µL, Solarbio). Color development was stopped after 15 minutes with Elisa Stop Solution (Solarbio), and the plates were read in a Thermo ScientificTM VarioskanTM LUX at a 450 nm wavelength.

### 4.14 Serum neutralization assays

All serum samples were inactivated by incubation in a water bath at 56°C for 30 minutes. The inactivated serum was then subjected to a 3 - fold serial dilution in a 96 - well plate using 2% FBS (Sigma - Aldrich) DMEM (Gibco), with dilutions of 1:20, 1:60, 1:180, 1:540, 1:1620, and 1:4860. A virus stock with a previously determined titer, stored at -80°C, was diluted to 2666 TCID50/mL (200 TCID50/75 µL) using 2% FBS DMEM. One hundred microliters of the diluted serum was added to each well of the 96 - well plate, followed by the addition of 100 µL of the working virus solution. The mixture was then shaken to homogenize and incubated at 37°C for 2 hours. Two replicate wells were prepared for each dilution. After further incubation in the incubator for 60 hours, the results were assessed by observing cytopathic effects, and images were captured using the PE High Content Imaging System (Operetta CLS).

### 4.15 Intracellular cytokine staining

Three weeks after the booster immunization, the BALB/c mice were euthanized, and their spleens were collected. Single-cell suspensions were prepared by grinding the spleens, and lymphocytes were isolated using density gradient centrifugation. 1 ml of lymphocytes from the experimental group mice was added to each well of a 24 - well plate, corresponding to 2 × 10^6 cells per well. RSV F peptides at a concentration of 2 μg/mL were added to each assay well and incubated for 1 - 2 hours. After stimulation, Protein Transport Inhibitor (containing Brefeldin A) was added to each well, followed by continued incubation for 10 hours. After discarding the supernatant and washing, Fixable Violet Stain 510 (BD Pharmingen) was added, and the mixture was vortexed to ensure uniformity. It was then incubated at room temperature in the dark for 15 minutes. Following two washes, Mouse BD Fc Block (BD Pharmingen) was added to block non - specific binding for 15 minutes at 4°C. Cells were stained with fluorescently - labeled antibodies specific to cell surface markers: FITC Hamster anti - Mouse CD3e, APC Rat Anti - Mouse CD4, and PerCP - CyTM5.5 Rat Anti - Mouse CD8a from BD Pharmingen at 4°C. After 30 minutes of incubation, cells were washed and incubated with 200 µL/well Fixation and Permeabilization Solution (BD Pharmingen) at 4°C for 20 minutes, washed twice with perm/wash buffer (BD Pharmingen), and stained with a cocktail of fluorescently labeled anti - cytokine antibodies (BV605 Rat Anti - Mouse IL - 2, PE - CyTM7 Rat Anti - Mouse IL - 4, BV421 Rat Anti - Mouse TNF, PE Rat Anti - Mouse IFN - γ, all from BD Pharmingen). The cells were washed once with perm/wash buffer and resuspended in 200 µL PBS. Samples were analyzed by flow cytometry (Beckman Cyto FLEX S), and the signal value for each item was subtracted by the signal value of the unstimulated group. Statistical comparison between groups was performed using an unpaired t - test with Welch’s correction via GraphPad Prism^®^ 8 software.

### 4.16 Focus forming assay

HEp - 2 cells with good growth status were seeded in a 96 - well plate and cultured overnight in an incubator at 37°C with 5% CO_2_. Mouse lung tissue was homogenized at 4°C and then centrifuged at 4000 rpm for 10 minutes at 4°C. The supernatant was serially diluted 4 - fold from well 1 to well 6 to infect HEp - 2 cells, with the original solution used in well 1. The 96 - well plate was placed in a plate centrifuge and centrifuged at 350 g for 30 minutes at 30°C. After that, the plate was incubated in a 37°C, 5% CO_2_ incubator for 1 hour. The culture medium in the wells was then replaced with maintenance medium containing 2% fetal bovine serum (FBS) (Sigma) and cultured in a 37°C, 5% CO_2_ incubator for 24 hours. After 24 hours of culture, the old culture medium was discarded. The wells were washed three times with PBS, followed by the addition of 100μL of 4% paraformaldehyde (Biosharp) per well, and fixed at room temperature overnight. The supernatant was discarded, and the wells were washed three times with PBS for 3 minutes each time. Then, 100μL of 0.1% Triton - X100 solution (Thermo Fisher) was added to each well for permeabilization at room temperature for 15 minutes. The supernatant was discarded again, and the wells were washed three times with PBS for 3 minutes each time. Next, 100μL of 5% BSA (ABCONE) - PBS solution was added to each well for blocking at room temperature for 1 hour. The supernatant was discarded, and the wells were washed three times with PBS for 3 minutes each time. The Respiratory Syncytial virus antibody (FITC) (Genetex) was diluted 1:100 with PBS and added to each well at 100μL per well, followed by incubation at 4°C overnight. The number of fluorescent spots in the cells was observed under a fluorescence inverted microscope, and the number of positive spots in each well was recorded.

### 4.17 Quantitative polymerase chain reaction

Total RNA was extracted from the lung homogenate of BALB/c mice and then diluted to a uniform concentration.The reaction system was set up using the HiScript^®^ II One Step qRT-PCR Probe Kit according to the manufacturer’s instructions. The specific primers for the RSV A2 F protein were synthesized as follows: Forward primer (5’-3’): CGAGCCAGAAGAGAACTACCA; Reverse primer (5’-3’): CCTTCTAGGTGCAGGACCTTA. The amplification reaction program was set on the qPCR instrument following the kit instructions, and the CT values were obtained. The copy numbers were calculated based on the standard curve.

### 4.18 Histological analysis

The right lobe of the lung from each BALB/c mouse was dissected and inflated with 10% neutral buffered formalin to restore its normal volume, then immersed in the same fixative solution. After fixation, the lungs were embedded in paraffin, sectioned, and stained with hematoxylin and eosin (H&E). A pathologist evaluated the H&E - stained slides for signs of peribronchiolitis (inflammatory cell infiltration around the bronchioles), perivasculitis (inflammatory cell infiltration around small blood vessels), interstitial pneumonia (inflammatory cell infiltration and thickening of alveolar walls), and alveolitis (presence of cells within alveolar spaces). The slides were scored on a severity scale ranging from 0 to 4.

## Data Availability

The original contributions presented in the study are included in the article/supplementary material. Further inquiries can be directed to the corresponding authors.
